# Primary Giant Mediastinal Hydatid Cyst Extending to the Superior Thoracic Inlet

**DOI:** 10.1590/0037-8682-0176-2022

**Published:** 2022-09-19

**Authors:** Yener Aydin, Ali Bilal Ulas, Atilla Eroglu

**Affiliations:** 1 Ataturk University, Medical Faculty, Department of Thoracic Surgery, Erzurum, Turkey.

**FIGURE 1:**
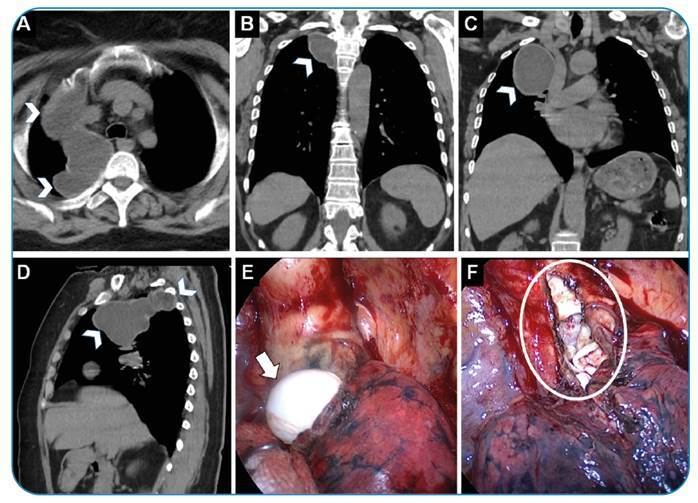
**(A)** Axial, **(B, C)** coronal, and **(D)** sagittal thoracic computed tomography sections show a mediastinal cystic lesion of approximately 10 x 4 cm (arrowheads) extending from the apex to the perihilar area in the right hemithorax. **(E, F)** The lesion was diagnosed intraoperatively as a hydatid cyst (arrow, laminated membrane; circle, cyst cavity).

A 57-year-old woman presented to our hospital with chest and shoulder pain. A giant mediastinal cystic lesion extending from the apex to the inferior perihilar area on the right hemithorax was detected radiologically. The patient underwent video-assisted thoracoscopic surgery. The intraoperative lesion was identified as a hydatid cyst and resected thoracoscopically (Figure 1). 

Hydatid cysts caused by metacestode forms of *Echinococcus granulosus* are commonly located in the liver and lungs. The mediastinal involvement rate is 0.61%, and primary mediastinal localization is less common^1^. Symptoms depend on the cyst size, location, and compression on adjacent structures, such as the esophagus, aorta, trachea, vena cava, and phrenic nerve^2^ ^,^ ^3^. Although primary giant mediastinal hydatid cysts are rare, they should be considered in the differential diagnosis of all mediastinal lesions.
